# The Outcomes of Myeloid Sarcoma in 64 Pediatric Patients and the Impact of Allogeneic Hematopoietic Stem Cell Transplantation on Treatment Results

**DOI:** 10.3390/children13030343

**Published:** 2026-02-27

**Authors:** Magdalena Samborska, Jolanta Skalska-Sadowska, Jacek Wachowiak, Małgorzata Czogała, Walentyna Balwierz, Szymon Skoczeń, Natalia Bartoszewicz, Jan Styczyński, Tomasz Ociepa, Tomasz Urasiński, Grażyna Wróbel, Krzysztof Kałwak, Katarzyna Muszyńska-Rosłan, Anna Szmydki-Baran, Iwona Malinowska, Paweł Łaguna, Agnieszka Mizia-Malarz, Renata Tomaszewska, Tomasz Szczepański, Agnieszka Chodała-Grzywacz, Grażyna Karolczyk, Lucyna Maciejka-Kembłowska, Marta Kozłowska, Ninela Irga-Jaworska, Katarzyna Mycko, Wanda Badowska, Katarzyna Bobeff, Wojciech Młynarski, Radosław Chaber, Joanna Zawitkowska, Katarzyna Drabko, Katarzyna Derwich

**Affiliations:** 1 Department of Pediatric Oncology, Hematology and Transplantology, Poznan University of Medical Sciences, 60-572 Poznan, Poland; jskalska@skp.ump.edu.pl (J.S.-S.); jacek.wachowiak@ump.edu.pl (J.W.); kderwich@ump.edu.pl (K.D.); 2 Department of Pediatric Oncology and Hematology, Institute of Pediatrics, Jagiellonian University Medical College, 30-663 Krakow, Poland; malgorzata.czogala@uj.edu.pl (M.C.); walentyna.balwierz@uj.edu.pl (W.B.); szymon.skoczen@uj.edu.pl (S.S.); 3 Department of Pediatric Hematology and Oncology, Collegium Medicum, Nicolaus Copernicus University Torun, 85-094 Bydgoszcz, Poland; natalabar@wp.pl (N.B.); jstyczynski@cm.umk.pl (J.S.); 4 Department of Pediatrics and Hemato-Oncology, Pomeranian Medical University, 70-001 Szczecin, Poland; tomasz.ociepa@pum.edu.pl (T.O.); tomasz.urasinski@pum.edu.pl (T.U.); 5 Clinical Department of Pediatric Bone Marrow Transplantation, Oncology and Hematology, Wroclaw Medical University, 20-529 Wroclaw, Poland; grazyna.wrobel@umw.edu.pl (G.W.); krzysztof.kalwak@umed.wroc.pl (K.K.); 6 Department of Pediatric Oncology and Hematology, Medical University of Bialystok, 15-274 Białystok, Poland; katarzyna.muszynska-roslan@umb.edu.pl; 7 Department of Pediatrics, Oncology, Hematology and Transplantology, Medical University of Warsaw, 02-091 Warsaw, Poland; aszmydki@tlen.pl (A.S.-B.); iwona.malinowska@wum.edu.pl (I.M.); pawel.laguna@wum.edu.pl (P.Ł.); 8 Department of Oncology, Hematology and Chemotherapy, Upper Silesia Children’s Care Health Centre, Medical Univeristy of Silesia, 40-752 Katowice, Poland; amizia@gczd.katowice.pl; 9 Department of Pediatric Hematology and Oncology, Zabrze, Medical University of Silesia, 41-800 Katowice, Poland; rtomaszewska@szpital.zabrze.pl (R.T.); szczep57@poczta.onet.pl (T.S.); 10 Department of Pediatric Hematology and Oncology, Regional Polyclinic Hospital in Kielce, 25-734 Kielce, Poland; aga.chodala@vp.pl (A.C.-G.); grazyna.karolczyk@wszzkielce.pl (G.K.); 11 Department of Pediatrics, Hematology and Oncology, Medical University of Gdansk, 80-952 Gdańsk, Poland; lucynamaciejka@o2.pl (L.M.-K.); marta.kozlowska@gumed.edu.pl (M.K.); ninela.irga-jaworska@gumed.edu.pl (N.I.-J.); 12 Department of Pediatrics and Hematology and Oncology, Province Children’s Hospital, 10-561 Olsztyn, Poland; katarzynamycko@tlen.pl (K.M.); hematologia@wssd.olsztyn.pl (W.B.); 13 Department of Pediatrics, Oncology and Hematology, Medical University of Lodz, 91-738 Lodz, Poland; katarzyna.bobeff@umed.lodz.pl (K.B.); wojciech.mlynarski@umed.lodz.pl (W.M.); 14 Department of Pediatrics, Faculty of Medicine, University of Rzeszow, Clinic of Pediatric Oncology and Hematology, 35-959 Rzeszow, Poland; rchaber@ur.edu.pl; 15 Department of Paediatric Haematology and Oncology and Transplantology, Medical University of Lublin, 20-093 Lublin, Poland; joanna.zawitkowska@umlub.pl (J.Z.); katarzyna.drabko@umlub.pl (K.D.)

**Keywords:** myeloid sarcoma, myeloid leukemia, children

## Abstract

**Background:** Myeloid sarcoma (MS) is a malignant extramedullary tumor that occurs in patients with acute myeloid leukemia (AML), myelodysplastic syndrome (MDS), or chronic myeloid leukemia (CML). The standard first-line treatment for MS is intensive chemotherapy according to the AML protocol, regardless of bone marrow involvement. The role of allogeneic hematopoietic stem cell transplantation (alloHSCT) in the treatment of pediatric patients with MS requires further investigation. The aim of the study was to evaluate treatment outcomes for MS in pediatric patients with a focus on assessing the impact of allogeneic hematopoietic stem cell transplantation (alloHSCT) on treatment efficacy. **Material and Methods:** The study included 64 patients aged 0 to 19 years from 15 pediatric oncology centers in Poland who were diagnosed with MS between 1998 and 2024. An Excel database was created to collect data on clinical features and treatment methods and outcomes. **Results:** The probability of 5-year overall survival (pOS) for the entire cohort was 0.63 ± 0.07, while the 5-year event-free survival (pEFS) and 5-year relapse-free survival (pRFS) were 0.62 ± 0.07 and 0.72 ± 0.07, respectively. Treatment outcomes were compared between patients who underwent allogeneic hematopoietic stem cell transplantation (alloHSCT) in first complete remission (ICR) (n1 = 17/64; 27%) and those who did not receive alloHSCT (n2 = 47/64; 73%). In the alloHSCT group (n1), the estimated survival probabilities were pOS = 0.49 ± 0.13, pEFS = 0.44 ± 0.14, and pRFS = 0.40 ± 0.14. In the non-alloHSCT group (n2), these values were pOS = 0.68 ± 0.08, pEFS = 0.68 ± 0.08, and pRFS = 0.84 ± 0.06. The difference in pRFS between groups n1 and n2 was statistically significant (*p* = 0.0049). Extramedullary relapses were more frequently observed in patients who had undergone allogeneic hematopoietic stem cell transplantation (alloHSCT) (*p* = 0.0001). **Conclusions:** Allogeneic hematopoietic stem cell transplantation (alloHSCT) does not improve the outcome of patients with MS. Further research is needed to identify effective strategies for sustaining remission in patients with MS after alloHSCT.

## 1. Introduction

Myeloid sarcoma (granulocytic sarcoma, myeloid tumor, chloroma) is a neoplasm composed of immature or mature myeloid blast cells [1]. Most commonly, it is associated with acute myeloid leukemia, clinically presenting as an extramedullary located mass. However, it may also precede bone marrow infiltration or present as an extramedullary relapse [1,2]. The latter is more frequently observed following allogeneic bone marrow transplantation [2]. MS may also occur in patients with blastic phase of transformed myeloproliferative neoplasms (MPNs), myelodysplastic syndromes (MDS), or MDS/MPNs [1,2,3,4]. The World Health Organization in 2008 defined myeloid sarcoma as an extramedullary proliferation of myeloid cells that disrupts the surrounding tissue architecture. This distinction clarifies the difference between myeloid sarcoma and extramedullary disease, the latter being a broader entity that includes, for example, central nervous system involvement. According to the latest WHO and International Consensus Classification (ICC) published in 2022, the definition of myeloid sarcoma remains unchanged [3].

The incidence of MS in adults varies from 0.2 to 2.8%; however, many authors suggest that the data may be underestimated, as patients with newly diagnosed AML are not routinely screened for extramedullary tumor unless they exhibit symptoms [2]. There is more data about post-allo-HSCT MS, in which the incidence was reported at 5–12%, which is 7–46% of total relapses [3]. In children, the prevalence of MS reported in the literature reaches up to 40%; however, studies including a large number of pediatric patients are lacking [5,6].

The “gold standard” to establish the diagnosis of MS is a biopsy of the tumor and immunohistochemical analysis [5,6]. The panel of antibodies should consist of the following: MPO, CD14, CD68, lysozyme, CD117, CD11c, CD13, CD33. The inclusion of CD20, CD79a, CD3 and CD45RO helps to exclude B-cell and T-cell lymphomas, which is crucial, as T-cell lymphoma is the most common misdiagnosis [5,6,7]. In cases with concomitant bone marrow involvement, the decision to perform a tumor biopsy depends on the patient’s condition and tumor location. If the biopsy is unsafe, the diagnosis may be based on imaging techniques, including positron emission tomography (PET), whose role in the diagnostic approach in MS has been well established [3,8]. Cytogenetics and molecular examinations from bone marrow and, if available, from tumor tissue, are also vital to establish an accurate diagnosis, as genetic abnormalities may influence treatment strategies [1,6].

The therapy of myeloid sarcoma in children is based on intensive chemotherapy according to the AML protocol [1]. The role of alloHSCT in children with MS remains a subject of ongoing debate, with conflicting findings reported in various studies. Here, we report the results of the study including a large cohort of pediatric patients with MS, making this report unique within the literature on MS in children, which is predominantly composed of case reports.

## 2. Material

The study included 64 patients aged 0 to 19 years (29 girls, 35 boys, mean age 7.9, median age 7.5 years), from 15 pediatric oncology centers in Poland, diagnosed with MS between 1998 and 2024. The diagnosis of MS was established in accordance with the World Health Organization (WHO) classification, which defines myeloid sarcoma as an extramedullary proliferation of myeloid cells disrupting the normal tissue architecture (WHO 2008, 2022) [3].

Biopsy and immunohistochemistry were performed on 42 patients. In 22 patients, the diagnosis of MS was established based on bone marrow assessment and imaging techniques. Treatment response was assessed primarily using imaging modalities, including positron emission tomography (PET-CT) [5].

## 3. Methods

Clinical, therapeutic, and outcome-related data were systematically recorded in a purpose-built spreadsheet developed in Microsoft Excel. For the purpose of analysis, an event was defined as any of the following: relapse of disease, progression, death from any cause, or diagnosis of a secondary malignancy.

Response to treatment was determined according to the respective AML treatment protocol. The categories of response included complete remission (CR), partial remission (PR), and late remission (LR). Importantly, PR was assigned in cases demonstrating partial tumor reduction, even when bone marrow findings fulfilled the criteria for complete remission.

Descriptive statistics for categorical variables were expressed as frequencies and corresponding percentages. Associations between categorical variables were evaluated using either the chi-square test or Fisher’s exact test, depending on data distribution and group size. Time-to-event outcomes—overall survival (OS), relapse-free survival (RFS), and event-free survival (EFS)—were analyzed using the Kaplan–Meier estimator, and survival probabilities (pOS, pRFS, pEFS) were calculated accordingly. Comparisons of survival distributions according to myeloid sarcoma (MS) manifestation and administration of allogeneic hematopoietic stem cell transplantation (alloHSCT) were performed with the log-rank test. All statistical computations were conducted using MedCalc software (version 20.123; MedCalc Software, Ostend, Belgium). Statistical significance was defined as a two-sided *p*-value below 0.05.

The study protocols were approved by the Ethics Committee of Medical University of Poznań, resolution number 455/15. All of participants have written consent to participate and publish the data. All procedures performed in studies involving human participants were in accordance with the ethical standards of the institutional and/or national research committee and with the 1964 Helsinki declaration and its later amendments or comparable ethical standards. Written informed consent for participation and publication was obtained from all participants aged 16–19 years. For participants younger than 16 years, written informed consent was obtained from a parent or legal guardian.

## 4. Results

### 4.1. Study Group Characteristics

In the study group, patients with AML predominated (49); thirteen patients had isolated MS without bone marrow involvement, one patient was diagnosed with CML, and one patient was diagnosed with MDS. The distribution of the study group based on the bone marrow disease diagnosis is presented in Table 1.

**Figure 1:**
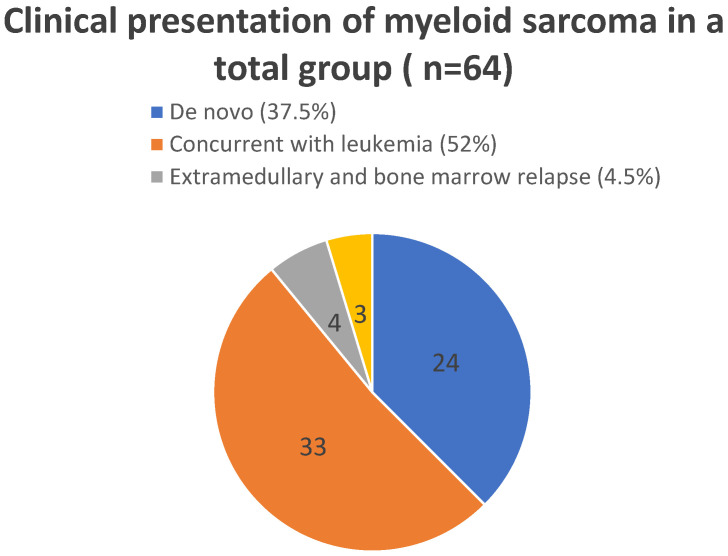
Clinical presentations of myeloid sarcoma depending on the time relation to bone marrow involvement.

Clinical presentations of myeloid sarcoma depending on the time relation to bone marrow involvement are illustrated in Figure 1. “De novo” cases include both patients with isolated myeloid sarcoma and patients who later developed bone marrow involvement during disease course. In these patients, myeloid sarcoma preceded leukemia. The time to development of bone marrow disease ranged from three to four weeks to four months.

The most frequently affected location was the skin, identified in 22 out of 64 cases (34%). The orbit was the second-most common localization (16/64, 25%). Other anatomical sites of lesions included the central nervous system, kidney, lung, liver, abdominal cavity and pelvis, mediastinum, maxillary, sphenoid, and ethmoid sinuses, pyramid of the temporal bone, soft tissues of the head and face, paraspinal mass, soft tissue of the forearm, and breast. Twenty patients (20/64, 31%) presented multifocal lesions (more than one).

Available genetic results of bone marrow in the study group are presented in Supplementary Material S1.

Almost all patients (63/64, 98%) received systemic chemotherapy. In twenty-seven patients (27/64, 42%), radiotherapy was administered as a part of the treatment, seven patients (7/64, 11%) underwent surgical treatment. Seventeen patients (17/64, 21%) were treated with alloHSCT.

### 4.2. Treatment Outcomes

**Figure 2:**
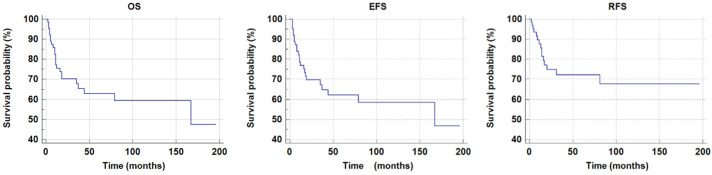
The 5-year overal survival probability (pOS), relapse-free survival (pRFS) and event-free survival probability (pEFS) in the total study group.

In the study group, CR was achieved in fifty-one patients (51/64, 79%). Among these, thirteen patients relapsed (13/51, 25%). Forty-one patients are alive (41/64, 64%) Twenty-three patients died (23/64, 36%); the major cause of death was disease progression (19/23, 83%), and four patients (4/23, 17%) died due to treatment complications. The mean overall survival (OS) was 121.3 ± 12.1 months, and the 5-year overall survival probability was 0.63 ± 0.07. The estimated OS rates were 75.5% at one year and 70.3% at two years, remaining stable through thirty months. The mean EFS was 119.4 ± 12.3 months, with a 5-year EFS probability of 0.62 ± 0.07. The EFS rates were 76.6% at one year and 69.4% at two years, with no further decline observed up to thirty months. Relapse occurred in 15 patients. The mean relapse-free survival (RFS) was 140.1 ± 12.3 months, and the 5-year RFS probability was 0.72 ± 0.06. The RFS rates were 87.7% at one year, 74.9% at two years, and 72.3% at thirty months, indicating a gradual decline over time. Figure 2 shows pOS, pRFSm, and pEFS curves.

Treatment outcomes were compared between patients who underwent allogeneic hematopoietic stem cell transplantation (alloHSCT) in first complete remission (ICR) (n1 = 17/64; 27%) and those who did not receive alloHSCT (n2 = 47/64; 73%).

In the n1 group, death occurred in eight out of seventeen patients. The mean survival time was 79.39 ± 16.61 months (mean ± SE). The 5-year survival probability was pOS = 0.49 ± 0.13. In the n2 group, death occurred in 15 out of 47 patients. The mean survival time was 128.85 ± 13.95 (months) (mean ± SE). The 5-year survival probability was pOS = 0.68 ± 0.08. No statistically significant differences were observed (*p* = 0.3177, log-rank test). The 5-year event-free survival probability (pEFS) in groups n1 and n2 was n1 pEFS = 0.44 ± 0.14 and n2pEFS = 0.68 ± 0.08, respectively. No statistically significant difference was observed (*p* = 0.1974, log-rank test).

**Figure 3:**
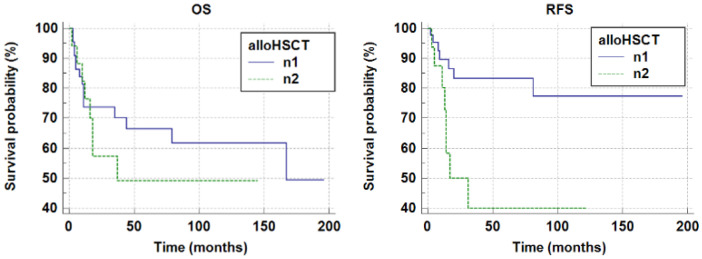
The 5-year overall survival probability and the 5-year relapse-free survival probability in groups n1 and n2 (n1 pOS 0.49 ± 0.13; n2 pOS 0.68 ± 0.08, *p* = 0.3177; n1 pRFS = 0.40 ± 0.14, n2 pRFS =0.84 ± 0.06, *p* = 0.0049).

In group n1, relapse occurred in eight out of seventeen patients. The mean relapse-free survival time was 57.61 ± 14.97 months (mean ± SE). In group n2, mean relapse-free survival time was 160.14 ± 12.48 months (mean ± SE). The 5-year relapse-free survival probabilities (pRFS) were as follows: n1pRFS = 0.40 ± 0.14, n2pRFS = 0.84 ± 0.06. A statistically significant difference was observed (*p* = 0.0049). Data are demonstrated in Table 2. Figure 3 shows pOS and pRFS curves of group n1 and n2. All relapsed patients in group n1 (8/8) presented extramedullary relapse; in 5/8 cases the relapse was combined with bone marrow relapse. Extramedullary relapse was more frequently observed in patients who had undergone allogeneic hematopoietic stem cell transplantation (alloHSCT) (*p* = 0.0001).

The outcomes of two clinical presentations of MS were compared—patients with de novo MS (24 patients) and those with MS concurrent with leukemia (33 patients). The 5-year overall survival and event-free survival probabilities were significantly higher in patients with myeloid sarcoma (MS) presenting concurrently with leukemia than in patients with de novo MS (OS: 0.82 ± 0.07 vs. 0.50 ± 0.11, *p* = 0.0037; EFS: 0.79 ± 0.08 vs. 0.50 ± 0.10, *p* = 0.0097).

## 5. Discussion

The prognosis for adult patients with myeloid sarcoma is poor [3,9]. The prognostic significance of MS in children remains unclear. Some authors demonstrated that myeloid sarcoma in children, except for the cutaneous form, is associated with a better prognosis [1,10,11]. More recent studies have demonstrated that the presence of extramedullary manifestation at diagnosis is associated with poorer outcomes. Li et al. showed that extramedullary infiltration present at diagnosis is an adverse prognostic factor, associated with shorter event-free survival (EFS) and overall survival (OS) [12]. Stove et al. analyzed 73 children with extramedullary leukemia (EML). The term EML in their study encompassed both central nervous involvement and myeloid sarcoma. Their findings indicated that 5-year overall survival (OS) was significantly lower in the EML group (64% vs. 73%, *p* = 0.04) [13]. Treatment results of myeloid sarcoma patients were as follows: 5-year EFS 57% and 5-year OS 68% which are similar to the results presented in our study [13]. In the analysis of Xu LH et al., MS in children was associated with a low complete remission rate, high induction death, poor 5-year EFS, and OS. Interestingly, the authors also showed that KMT2A rearrangement had a negative impact on clinical outcomes in AML patients with MS [14].

In our study, patients with de novo myeloid sarcoma had significantly lower pOS and pEFS than patients with MS concomitant with bone marrow disease. Little is known about the impact of the clinical presentation of MS on treatment results in children. In Zhao’s analysis, which included both adults and children, patients with primary MS (without bone marrow involvement) had significantly lower pOS compared to patients with MS and intramedullary disease [9]. Zipin Xing et al. categorized patients with MS into two subgroups: patients with a hematopoietic location of the tumor (lymph nodes, liver and spleen) and those with tumor in other locations. Patients with non-hematopoietic MS and a clinical presentation of MS as the primary malignancy exhibited a higher survival rate [15]. Better outcomes were observed in children than in adults. Interestingly, the authors developed nomograms to predict prognosis in patients with non-hematopoietic locations of MS [15]. Data about the prognostic significance of isolated extramedullary relapse after alloHSCT are also unclear. In Yuda’s analysis, patients with isolated extramedullary post-transplant relapse presented better 2-year overall survival than those with combined bone marrow and extramedullary relapse or bone marrow relapse [16].

It is well documented that systemic chemotherapy is a frontline treatment for myeloid sarcoma, both in children and adults, regardless of bone marrow involvement. However, there are still a lot of questions about the optimal therapeutic strategy in MS, as treatment results remain insufficient. Data about the role of alloHSCT in myeloid sarcoma are conflicting. Chevalier et al. recommend allogeneic hematopoietic stem cell transplantation after chemotherapy in both isolated myeloid sarcoma and myeloid sarcoma concurrent with leukemia [17]. Shan et al. also reported that alloHSCT improved the outcomes of patients with MS who achieved remission after chemotherapy [18]. In Zhao et al.’s analysis, patients who underwent alloHSCT presented significantly better median survival time than patients treated with local therapy or intensive chemotherapy alone [9]. The authors suggest that maintenance therapy with decitabine after alloHSCT may help to sustain remission in patients with MS [9]. However, some authors demonstrated that alloHSCT had no significant effect on the survival of AML patients with MS [12,14]. In our analysis, patients who underwent allogeneic hematopoietic stem cell transplantation (alloHSCT) had a significantly lower probability of relapse-free survival compared to those who did not undergo transplantation. It has already been documented that after alloHSCT, the frequency of extramedullary relapse is higher. One potential explanation for the increased risk of extramedullary recurrence following alloHSCT is the reduced graft-versus-leukemia (GvL) effect in extramedullary lesions [3,19]. The reason for this is diminished immunologic surveillance in extramedullary tissues, as patrolling donor T cells and natural killer (NK) cells are mainly concentrated in the bone marrow [9,19,20]. This means that the effect of chronic graft-versus-host disease (GvHD) does not have a protective effect against relapse in the extramedullary sites, what has also been demonstrated in Hazar et al.’s report [19,20,21]. The authors also reported that extramedullary disease before transplantation was an independent risk factor associated with an increased incidence of extramedullary relapse [21].

In this context, a novel therapy approach is needed for patients with MS. Li et al. suggest that gemtuzumab ozogamicin (GO) may improve the outcome of patients with MS, contrary to alloHSCT [12]. Piccaluga et al. also concluded that GO may be an effective agent in the treatment of MS [22]. Xue Zhang et al. reported two cases of adult patients with MS who underwent microtransplantation. The procedure is an alternative method of allotransplantation, with a low toxicity, but it may retain part of the graft-versus-leukemia (GVL) effect. Both patients achieved durable disease control [23].

An increasing amount of data in the literature highlights the role of hypomethylating agents and venetoclax in the treatment of myeloid sarcoma [24,25,26]. These agents should also be considered as maintenance therapy after alloHSCT is performed as consolidation therapy in patients with MS [1].

In MS associated with AML harboring a mutation of fms-like tyrosine kinase 3 (FLT3) gene, FLT3 inhibitors might be effective. There are a few reports of successful treatment with gliteritinib in adult patients with MS [27,28,29]. Some data supporting the use of IDH inhibitors in the treatment of MS exist, as IDH1/IDH2 mutations have been described in MS [3]. Sonedeb et al. documented good response in three out of four patients with IDH mutation detected in the tissue of the extramedullary involved site [30].

There is still much to be investigated regarding new drugs used in AML in the context of myeloid sarcoma. Little is known about the efficacy of CPX 351 against extramedullary sites. Gianfelici et al. reported a case of successful treatment with CPX 351 in an adult woman with MS located in the skin [31]. Further research is also needed to determine the role of menin inhibitors in the treatment of myeloid sarcoma with KMT2A rearrangement.

The use of menin inhibitors may represent a breakthrough in the treatment of myeloid sarcoma with KMT2A rearrangement [32].

## 6. Conclusions

Allogeneic hematopoietic stem cell transplantation (alloHSCT) does not improve the outcome of patients with MS. Moreover, patients who have been transplanted have a lower 5-year probability of relapse-free survival, indicating a diminished graft-versus-leukemia (GvL) effect in extramedullary sites. Further research is needed to identify effective strategies for maintaining remission in MS patients following allo-HSCT. Additionally, future studies should focus on the genetic characteristics of MS, as this may facilitate the development of novel treatment approaches.

## 7. Limitations

This study has limitations, as data about the occurrence of GvHD, type of conditioning, and type of donor in transplanted patients are lacking. Future research should include analysis with accurate data about the transplantation procedure in patients who underwent alloHSCT.

In addition, due to the multicenter and retrospective design of the study, some cytogenetic and molecular data were unavailable.

## Figures and Tables

**Table 1:** Distribution of the study group based on the bone marrow disease diagnosis.

Bone Marrow Disease Diagnosis	Number of Patients (n)
**AML**	49 (77%)
CML *	1 (1.5%)
**MDS**	1 (1.5%)
**Isolated Myeloid Sarcoma** **(without bone marrow involvement)**	13 (20%)

* CML—chronic myeloid leukemia.

**Table 2:** Outcome of the treatment in the group of patients who received allogeneic hematopoietic stem cell transplantation (alloHSCT)—**group n1**—and those who did not received alloHSCT—**group n2**.

	pOS	pEFS	pRFS
n1 = 17 (with alloHSCT)	0.49 ± 0.13	0.44 ± 0.14	0.40 ± 0.14
n2 = 47 (without alloHSCT)	0.68 ± 0.08	0.68 ± 0.08	0.84 ± 0.06

## Data Availability

The data analyzed in this study is subject to the following licenses/restrictions: The dataset was created by the author on the basis of clinical data and patient history available in pediatric oncology centers. The author has access to the data, which are not public. Requests to access these datasets should be directed to magdalena.samborska@ump.edu.pl. This retrospective study involved analysis of anonymized patient data and did not require ethical approval, in accordance with institutional and national guidelines.

## Supplementary Materials

The following supporting information can be downloaded at: https://www.mdpi.com/article/10.3390/children13030343/s1, SI: The results of all genetic tests of bone marrow in patients from the study group (*n* = 64).
