# Supplementary material for: The Outcomes of Myeloid Sarcoma in 64 Pediatric Patients and the Impact of Allogeneic Hematopoietic Stem Cell Transplantation on Treatment Results
Source: Children (Basel). 2026 Feb 27;13(3):343. doi: 10.3390/children13030343 (PMC13025773; doi:10.3390/children13030343)
Supplement: Supplementary file 1 [file children-13-00343-s001.zip › children-4099064-supplementary.pdf]

Supplementary material S1.

The results of all genetic tests of bone marrow in patients from the study group (n=64).

No.	Genetic results
1	t8;21 (q22;q22)
2	RUNX1: RUNX1T1 [t(8;21)(q22;q22.1) (bone marrow)
3	No data.
4	46,XY,inv(3)(p?21q?12)[21].nuc ish(hTERT.EGR1,CSF1R)x2[200],(D7Z1,D8Z2)x2[200],(CUTL1,D7S2419/D7S688/D7S2640)x2[200].(TP53,D17Z1)x2[200]
5	47,XX,+21
6	No data.
7	Karyotype: 90–92,XXYY,der(4)[15].<br>M-BCR/ABL – negative; MLL/AF4 – negative; TEL/AML1 – negative; AML/ETO – negative; m-BCR/ABL – negative; E2A/PBX1 – negative; CBFβ-MYH11 – negative; FLT3-ITD – negative.
8	Karyotype: 45,X,−Y,t(8;21)[14] / 46,XY[3].<br>RT-PCR analysis: M-bcr/abl (−), m-bcr/abl (−)
9	t(8;21)
10	t (8,21)
11	t (8,21)
12	Any abnormalities
13	Any abnormalities
14	No data.
15	t(8,21)
16	t(8,21)
17	No data.
18	No data.
19	No data.
**20**	47,XX,del(3)(q22q24), del(12)(p13.1),+19[19]/46,XX [1]
21	KMT2A/MLLT3
22	46 XX , t(9,11), KMT2A:MLLT3
23	46,XX,der(9)t(9;11;17)(q12;q23;q25),der(11)t(9;11)(p22?;q23),der(17)t(9;17)(q12;q23), del(17)(q23q25).<br>FLT3 (-) In 70% of analysed cells MLL rearrangement was observed.
24	No data.
25	No data.
26	FLT3(-), WT1(-) NPM1 (-), Hema-Vision 28 neg (28 most common chromosomal rearrangements in AML)

27	46,XX,t(9;11)(9pter~9p22::11q23~11q14.2::9p22~9q32::11q23~11qter;11pter~ 11q14.2::11q2311q23::9q32~9qter)[4]/46,XX[16]
28	t(9;11)(p22;q23)
29	10% of cells with MLL rearrengement
30	No data.
31	No data.
32	No data.
33	No data.
34	No data.
35	47,XY,+8,t(11;19)(q23.3;p13.1)
36	46,XY,t(8;21)(q22;q22)[15]/ 46,XY[5]
37	No data.
38	fuzja KMT2A::MLLT3, 51,XX,+4,-6+8,+9,t(p21;q23),+12,+18,+mar[20]/46,XX[2]
39	fuzja KMT2A::MLLT3, 44, XX, t(11;19)(q23;p13)
40	MLL rearrangement in 93% of the analyzed cells. translocation t(9;11)(p21;q23)
41	No data.
42	In all metaphases, the translocation t(8;21) with fusion of the AML1/ETO genes on chromosome 8 and loss of the Y sex chromosome were detected, which is characteristic of AML M2. Additionally, complex aberrations involving chromosomes 11 and 22 were identified.<br>BCR/ABL1 – negative. ITD-FLT3 – negative, NPM1 – negative, PML-RARA – negative, inv(16) – negative.
43	No PML/RARA gene fusion, was detected. The BCR/ABL gene fusion was detected in 13/200 interphase nuclei. No tandem duplication or D835 mutation in the FLT3 gene was detected
44	Karyotype: 46,XY.<br>Among 200 analyzed interphase nuclei, no AML1/ETO gene fusion, and thus no t(8;21) translocation, was detected. Among 200 analyzed interphase nuclei, no inversion of chromosome 16 was detected. No tandem duplication or D835 mutation in the FLT3 gene was detected. Among 200 analyzed interphase nuclei, no PML/RARA gene fusion, and thus no t(15;17) translocation, was detected.<br>NCN = 6400 copies of WT1 per 10^4 copies of ABL.
45	In 90% (180/200) of the analyzed interphase nuclei, a double AML1/ETO gene fusion, and thus the t(8;21) translocation, was detected. No PML/RARA gene fusion, and thus no t(15;17) translocation; no TEL/AML1 gene fusion, and thus no t(12;21) translocation; no deletions involving 11q23 or other MLL-related rearrangements; no tandem duplication or D835 mutation in the FLT3 gene; no deletion of the long arm of chromosome 5; no BCR/ABL1 gene fusion, and thus no t(9;22) translocation; and no DEK/NUP214 gene fusion, and thus no t(6;9) translocation, were detected.<br><br>Karyotype: 45,X,Y,t(8;21)(q22;q22).
46	In 19% of the analyzed tumor cells, three signals originating from the ABL1 and BCR genes were observed.
47	In 10% of the analyzed interphase nuclei, trisomy of chromosome 8 was detected. GTG cytogenetics showed a normal karyotype in all metaphases. In 40% of the interphase nuclei, trisomy of chromosome 8 was observed. NMYC amplification analysis – not detected.
48	Karyotype: 46,XX. BCR/ABL1 – negative, TEL/AML1 (t(12;21)) – negative, MLL (11q23) – negative, FLT3 – negative, inv(16) – negative, WT1 – positive.
48	Analysis of the 28 most common chromosomal rearrangements with prognostic significance in leukemias – the presence of the MLL-AFF1 fusion gene t(4;11)(q21;q23) was detected.
49	No data.
50	No data.
51	No data.
52	No data.
53	No data.
54	46XX t(9;11)
55	No data.
56	No data.

57	No data.
58	Complex karyotype with clonal evolution.
59	No data.
60	t(10,11)
61	t(8;21)
62	No data.
63	Negative.
64	No data.
